# An Osteosarcoma Stem Cell Potent Nickel(II)-Polypyridyl Complex Containing Flufenamic Acid

**DOI:** 10.3390/molecules27103277

**Published:** 2022-05-19

**Authors:** Ginevra Passeri, Joshua Northcote-Smith, Roshane Perera, Nikola Gubic, Kogularamanan Suntharalingam

**Affiliations:** School of Chemistry, University of Leicester, Leicester LE1 7RH, UK; gp243@leicester.ac.uk (G.P.); jns7@leicester.ac.uk (J.N.-S.); uvrrbp1@student.le.ac.uk (R.P.); ng193@student.le.ac.uk (N.G.)

**Keywords:** metallodrugs, nickel, osteosarcoma stem cells, necroptosis, bioinorganic chemistry

## Abstract

Apoptosis resistance is inherent to stem cell-like populations within tumours and is one of the major reasons for chemotherapy failures in the clinic. Necroptosis is a non-apoptotic mode of programmed cell death that could help bypass apoptosis resistance. Here we report the synthesis, characterisation, biophysical properties, and anti-osteosarcoma stem cell (OSC) properties of a new nickel(II) complex bearing 3,4,7,8-tetramethyl-1,10-phenanthroline and two flufenamic acid moieties, **1**. The nickel(II) complex **1** is stable in both DMSO and cell media. The nickel(II) complex **1** kills bulk osteosarcoma cells and OSCs grown in monolayer cultures and osteospheres grown in three-dimensional cultures within the micromolar range. Remarkably, **1** exhibits higher potency towards osteospheres than the metal-based drugs used in current osteosarcoma treatment regimens, cisplatin and carboplatin, and an established anti-cancer stem cell agent, salinomycin (up to 7.7-fold). Cytotoxicity studies in the presence of prostaglandin E2 suggest that **1** kills OSCs in a cyclooxygenase-2 (COX-2) dependent manner. Furthermore, the potency of **1** towards OSCs decreased significantly upon co-treatment with necrostatin-1 or dabrafenib, well-known necroptosis inhibitors, implying that **1** induces necroptosis in OSCs. To the best of our knowledge, **1** is the first compound to implicate both COX-2 and necroptosis in its mechanism of action in OSCs.

## 1. Introduction

Apoptosis is a programmed, morphologically distinct form of cell death [1]. The vast majority of chemotherapeutics in preclinical and clinical development kill cancer cells through apoptosis [2,3]. Non-apoptotic forms of cell death have been characterised, such as necrosis, necroptosis, autophagy, paraptosis, and ferroptosis, and their activation by exogenous agents could help overcome chemotherapeutic resistance, which is often intimately linked to apoptosis dysfunction [4]. Necroptosis is markedly different from apoptosis and is classified as a regulated inflammatory mode of cell death [5]. Necroptosis involves the formation of necrosomes, an amyloid-like fibrillar complex, consisting of receptor-interacting protein kinase 1 (RIP1), receptor-interacting protein kinase 3 (RIP3), and mixed-lineage kinase domain-like protein (MLKL) [6,7]. The necrosome, once assembled, activates a multitude of downstream signaling pathways, which ultimately lead to cell death. The downstream processes include reactive oxygen species (ROS) production, permeabilisation of the cell membrane, depolarisation of the mitochondrial membrane, and bioenergetics diminution [8,9]. The necroptosis signaling cascade utilises proteins that are largely independent of apoptosis, therefore cancer cells that exhibit resistance to apoptosis-inducers could be vulnerable to necroptosis-inducers [10]. Stem cell-like sub-populations within certain tumour types (such as breast cancer and osteosarcoma) are thought to be responsible for relapse and metastasis (the main cause of cancer-associated deaths) [11,12]. These cells are inherently apoptosis resistant and could be viable therapeutic targets for necroptosis-inducers [13,14].

Compared to the plethora of apoptosis-inducing small molecules reported to date, only a small fraction of necroptosis-inducers have been identified and validated, and only a handful of this class of compounds contain a metal [15]. Furthermore, only three families of metal complexes have been demonstrated to induce necroptosis in cancer stem cells (CSCs) in vitro. A series of osmium(II) and ruthenium(II) complexes comprising of para-cymene, dichloroacetate, and 4,7-diphenyl-1,10-phenanthroline ligands were shown to induce necroptosis in breast CSCs [16]. We reported a series of nickel(II)-polypyridyl complexes bearing dithiocarbamate ligands with promising anti-breast CSC and anti-osteosarcoma stem cell (OSC) cytotoxicity in monolayer and three-dimensional cell cultures [17,18]. The lead complex within this series, [Ni(*N*,*N*-diethyldithiocarbamate)_2_(1,10-phenanthroline)] induced breast CSC and OSC death by necroptosis [17,18]. The induction of necroptosis was proved using conventional assays that probed known morphological and phenotypical features associated with necroptosis as well as unbiased predictive functional genetic analysis (based on RNA interference) [17,18]. The latter showed that [Ni(*N*,*N*-diethyldithiocarbamate)_2_(1,10-phenanthroline)] acted in a similar way to shikonin, a well-established necroptosis-inducer [17]. More recently we reported a series of nickel(II)-polypyridyl complexes bearing the non-steroidal anti-inflammatory drugs (NSAIDs) naproxen and indomethacin, with micromolar potency towards breast CSCs [19]. Mechanistic studies showed that the nickel(II) complexes triggered breast CSC death via a cyclooxygenase-2 (COX-2)-dependent pathway and in a manner that is blocked by necroptosis inhibitors [19]. Here, we have sought to expand this promising class of necroptosis-inducing agents by incorporating flufenamic acid, an NSAID that has been successfully used as a chemopreventive in various cancer models [20,21]. It should be noted that there have been a large number of reports on the biological properties of metal-NSAID complexes over the last decade [22,23]; however their anti-OSC properties and their ability to induce necroptosis have been rarely investigated. A number of nickel(II) complexes bearing NSAIDs (including flufenamic acid) and polypyridyl ligands have also been structurally characterised and have shown promising DNA and serum binding properties [24,25,26]. Herein, we report the synthesis and characterisation of two novel nickel(II)-flufenamic acid complexes and provide insight into the anti-OSC potential and mechanism of action of the lead complex.

## 2. Results and Discussion

**Figure 1:**
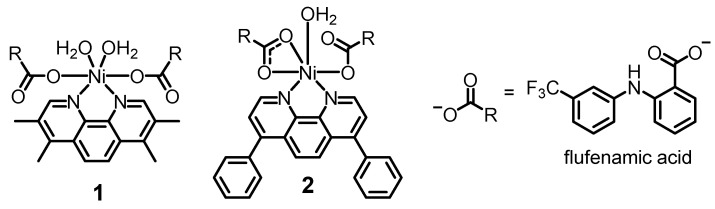
Chemical structures of nickel(II)-flufenamic acid complexes with 3,4,7,8-tetramethyl-1,10-phenanthroline or 4,7-diphenyl-1,10-phenanthroline (**1** and **2**) prepared and investigated in this study.

The chemical structures of the nickel(II)-flufenamic acid complexes, **1** and **2** prepared in this study are depicted in Figure 1. The nickel(II)-flufenamic acid complexes, **1** and **2**, were synthesised by reacting two equivalents of flufenamic acid (in the presence of potassium hydroxide) with one equivalent of NiCl_2_⋅6 H_2_O and the appropriate polypyridyl ligand (3,4,7,8-tetramethyl-1,10-phenanthroline for **1** and 4,7-diphenyl-1,10-phenanthroline for **2**) in methanol. The nickel(II)-flufenamic acid complexes, **1** and **2**, were obtained as pale green solids in reasonable yields (19–50%). The solid samples were subject to full characterisation using infra-red and UV-vis spectroscopy and elemental analysis (Figures S1 and S2). Analysis of the vibrational stretching frequencies associated to the asymmetric, ν_asym_(CO_2_) and symmetric, ν_sym_(CO_2_) carboxylato peaks provide insight into the binding mode of the carboxylic acid moiety in **1** and **2** to the nickel(II) centre [27,28]. The difference between the ν_asym_(CO_2_) and ν_sym_(CO_2_) carboxylato peaks for **1** and **2** (based on their ATR-FTIR spectra) was 205 cm^−1^ and 191 cm^−1^, respectively (Figure S1). This suggests a monodentate binding mode for the carboxylic acid moiety on flufenamic acid to the nickel(II) centre in **1** and a mixed monodentate-bidentate binding mode for **2** (as shown in Figure 1). The carboxylic acid group binding mode assignment for **1** and **2** is consistent with previous studies on structurally similar nickel(II)-polypyridyl complexes containing flufenamic acid and other NSAIDs [19,26]. The UV-vis spectra of **1** and **2** (50 µM) in PBS:DMSO (100:1) displayed intense signals between 277–288 nm associated to π–π* and metal-perturbed π–π* transitions originating from the flufenamic acid and 3,4,7,8-tetramethyl-1,10-phenanthroline or 4,7-diphenyl-1,10-phenanthroline units (Figure S2). The UV-vis spectra also displayed less intense, broader bands associated with high-energy metal-to-ligand charge-transfer (MLCT) and typical MLCT (d-π*) transitions at 300 nm and 340 nm for **1**, and 337 nm for **2**. The bulk purity and the chemical composition of **1** and **2** was analysed and confirmed by elemental analysis (see Experimental Section).

**Figure 2:**
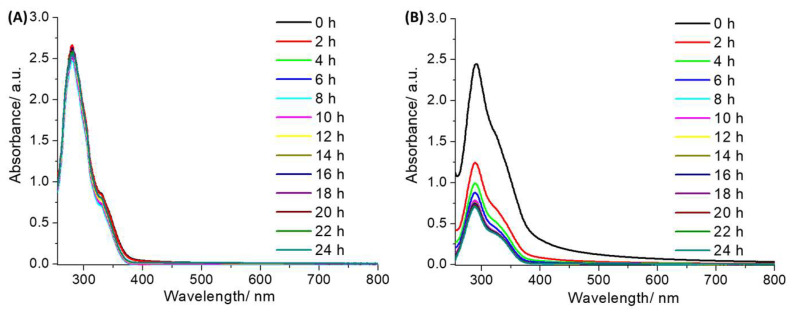
UV-Vis spectra of (**A**) **1** and (**B**) **2** (both 50 μM) in Dulbecco’s Modified Eagle Medium (DMEM):DMSO (200:1) over the course of 24 h at 37 °C.

The lipophilicity of a given compound provides valuable information on its likelihood of internalization by cells and its solubility in aqueous solutions. The lipophilicity of the nickel(II)-flufenamic acid complexes, **1** and **2**, was determined using the shake-flask method (the partition between octanol and water). The LogP value for **1** and **2** was 1.09 ± 0.06 and 1.70 ± 0.14, respectively. The LogP values for **1** and **2** are consistent with their relative structures; in other words, the nickel(II) complex bearing the bulkier and thus more hydrophobic polypyridyl ligand (4,7-diphenyl-1,10-phenanthroline) **2** exhibited a higher LogP value (than its analogue **1**). The LogP values for **1** and **2** imply that both nickel(II) complexes should be readily internalized by dividing cells. To investigate the stability of **1** and **2** in solutions used for cell-based studies, UV-vis spectroscopy studies were performed. Cell studies usually involve the preparation of stock solutions of the compound of interest in DMSO prior to dilution in cell culture media. Therefore, the UV-vis trace of **1** and **2** (50 µM) in DMSO and Dulbecco’s Modified Eagle Medium (DMEM):DMSO (200:1) was monitored over the course of 24 h. The π–π* and MLCT absorption bands associated to **1** and **2** remained unaltered in DMSO over the course of 24 h, which is indicative of good stability (Figures S3 and S4). This suggests that DMSO is a suitable solvent to use to prepare and store stock solutions of **1** and **2**. In DMEM:DMSO (200:1), the π–π* and MLCT bands for **1** were unchanged over the course of 24 h, suggestive of good stability under these conditions (Figure 2A). Under the same conditions, the absorbance of the π–π* and MLCT bands associated to **2** decreased by 72%; however, the wavelengths associated to the bands remained unchanged, suggestive of only modest stability (Figure 2B). Overall, these studies showed that the stability of **1** and **2** in cell culture media is heavily reliant on the nature of the polypyridyl ligand. The 3,4,7,8-tetramethyl-1,10-phenanthroline-bearing nickel(II) complex **1** was more stable than the 4,7-diphenyl-1,10-phenanthroline-bearing complex **2**. The difference in stability between **1** and **2** is likely to be related to the distinctive electronic and lipophilic influence of 3,4,7,8-tetramethyl-1,10-phenanthroline and 4,7-diphenyl-1,10-phenanthroline on the respective nickel(II) coordination complexes. Similar stability trends have been previously observed for structurally related metal(II)-NSAID complexes containing 3,4,7,8-tetramethyl-1,10-phenanthroline and 4,7-diphenyl-1,10-phenanthroline [19,29].

Monolayer cytotoxicity studies were performed to determine the potency of **1** towards bulk osteosarcoma cells and OSCs grown in two-dimensional cultures. Similar studies with **2** were not undertaken due to its instability in cell culture media (Figure 2B). Specifically, the potency of **1** towards OSC-depleted U2OS cells and OSC-enriched U2OS-MTX cells was determined using the MTT [3-(4,5-dimethylthiazol-2-yl)-2,5-diphenyltetrazolium bromide] assay. IC_50_ values, used to gauge potency, were derived from dose-response curves (Figure S5), and are summarised in Table 1. The nickel(II) complex **1** displayed micromolar potency towards both U2OS and U2OS-MTX cells, comparable to or more toxic than cisplatin and carboplatin, metal-based drugs used in frontline and secondary osteosarcoma treatments respectively [30]. The similar IC_50_ value of **1** towards U2OS and U2OS-MTX cells suggests that **1** has the capacity to kill both bulk osteosarcoma cells and OSCs with a single dose. Flufenamic acid was non-toxic towards both U2OS and U2OS-MTX cells at the concentration range tested (IC_50_ < 100 µM, Figure S6), implying that the monolayer cell toxicity imparted by **1** is largely independent of the flufenamic acid moiety. Notably, salinomycin, a well-known CSC-active compound, was significantly more toxic towards U2OS and U2OS-MTX cells than **1** [30].

**Figure 3:**
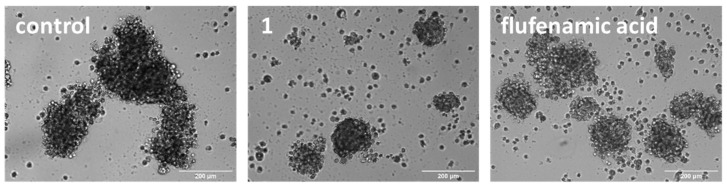
Representative bright-field images (×10) of U2OS-MTX osteospheres formed from single cell suspensions of U2OS-MTX cells without and with the addition of **1** or flufenamic acid (IC_20_ values) after 10 days incubation. Scale bar = 200 µm.

Three-dimensional cell cultures offer a more relevant model for assessing OSC potency and translation potential than monolayer cultures. U2OS-MTX cells grown in serum-free, low-attachment conditions form osteospheres (also called sarcospheres) which are an irregular collection of OSCs with naturalistic properties. The addition of **1** (at its corresponding IC_20_ value for 10 days) to single cell suspensions of U2OS-MTX cells markedly disrupted the formation of osteospheres compared to untreated control cells (Figure 3). Flufenamic acid also inhibited the formation of osteospheres but to a lesser extent than **1** (at its corresponding IC_20_ value upon 10 days incubation) (Figure 3). Under identical conditions, cisplatin and carboplatin (at their corresponding IC_20_ value) did not noticeably affect osteosphere formation, whereas salinomycin (at its corresponding IC_20_ value) did inhibit osteosphere formation but to a lesser degree than **1** [31]. Having shown that **1** is able to disrupt osteosphere formation, the colorimetric resazurin-based reagent, TOX8 was used to determine the effect of **1** on osteosphere viability. Interpolation of dose-response curves showed that **1** displayed low micromolar toxicity towards osteospheres (Table 1, Figure S7). Based on the IC_50_ values, **1** was 9.1-fold more potent towards three-dimensionally cultured osteospheres than U2OS-MTX cells grown in monolayer cultures. This is a remarkable result given that most small molecules exhibit lower potency towards spheroidal systems compared to monolayer systems (made up of the same cells). This trend was also evident for flufenamic acid, which was non-toxic towards U2OS-MTX cells grown in monolayer cultures but displayed micromolar potency towards osteospheres (IC_50_ = 13.17 ± 0.24 µM, Figure S7). This suggests that the flufenamic acid moiety may play a role in the osteosphere potency observed for **1**. Furthermore, **1** displayed significantly higher potency towards osteospheres than cisplatin, carboplatin, and salinomycin (up to 7.7-fold, *p* < 0.05) [30].

**Figure 4:**
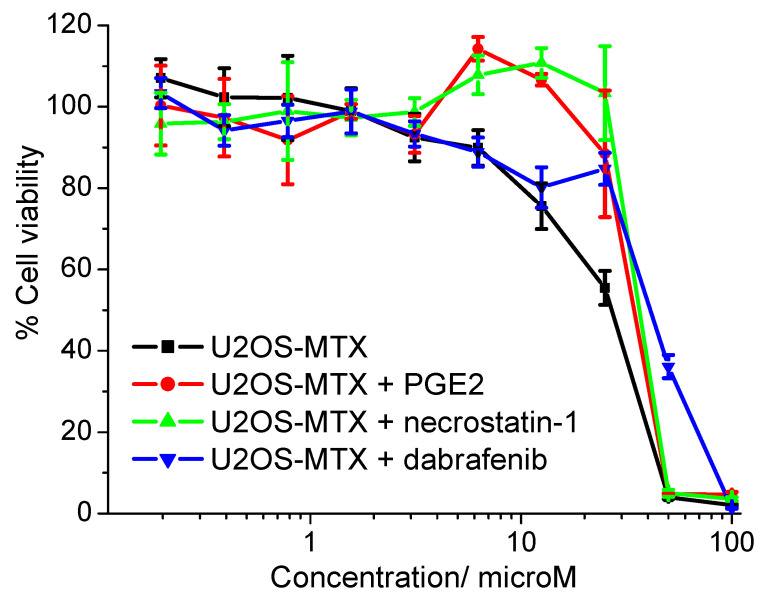
Representative dose-response curves for the treatment of U2OS-MTX cells with **1** in the absence and presence of PGE2 (20 µM), necrostatin-1 (20 µM), or dabrafenib (10 µM) after 72 h incubation.

Previously reported nickel(II)-polypyridyl complexes bearing NSAIDs were shown to induce breast CSC death by inhibiting COX-2 and triggering necroptosis [19]. To determine if the nickel(II)-flufenamic acid complex **1** induced a similar cellular response in OSCs, cytotoxicity studies were conducted with U2OS-MTX cells in the presence of specific inhibitors. The potency of **1** decreased significantly (*p* < 0.05) in the presence of the functional product of COX-2-catalysed arachidonic acid metabolism, prostaglandin E2 (PGE2) (20 µM) [32] and potent inhibitors of RIP1 and RIP3 (major components of necrosomes which are responsible for activating downstream necroptosis effectors), necrostatin-1 (20 µM) and dabrafenib (10 µM), respectively [33,34]. Specifically, the IC_50_ value of **1** towards U2OS-MTX cells increased to 34.21 ± 2.11 µM in the presence of PGE2, to 36.60 ± 0.57 µM in the presence of necrostatin-1, and to 41.06 ± 1.03 µM in the presence of dabrafenib (Figure 4). Co-incubation of **1** with caspase-dependent apoptosis inhibitor, z-VAD-FMK (5 µM) [35] and the unregulated necrosis inhibitor, IM-54 (10 µM) [36] did not lead to a statistically significant decrease in the potency of **1** towards U2OS-MTX cells. Specifically, the IC_50_ value of **1** towards U2OS-MTX cells increased marginally to 27.16 ± 0.48 µM in the presence of z-VAD-FMK and to 29.37 ± 1.61 µM in the presence of IM-54 (Figure S8). Collectively, this implies that **1** most probably kills OSC-enriched U2OS-MTX cells via a COX-2 dependent necroptosis pathway and not via an apoptosis or unregulated necrosis pathway.

## 3. Conclusions

In summary, we report the preparation and characterisation of two nickel(II) complexes, **1** and **2**, containing a bidentate polypyridyl ligand and two flufenamic acid moieties. The nickel(II) complex containing 3,4,7,8-tetramethyl-1,10-phenanthroline **1** was more hydrophilic and stable in cell culture media than the nickel(II) complex containing 4,7-diphenyl-1,10-phenanthroline **2**. This is consistent with the LogP values and solution stabilities reported for structurally related metal complexes containing polypyridyl ligands and NSAIDs. The 3,4,7,8-tetramethyl-1,10-phenanthroline-bearing complex **1** exhibited micromolar potency towards bulk osteosarcoma cells and OSCs grown in two-dimensional cultures. The potency was in the same range as cisplatin and carboplatin, albeit less than salinomycin. The equipotent nature of **1** towards bulk osteosarcoma cells and OSCs implies that it could, in theory, kill heterogeneous osteosarcoma populations containing differentiated and stem cell-like cells with a single micromolar dose. Strikingly, **1** was more potent towards three-dimensionally cultured osteospheres than OSCs cultured in monolayers. This is an unusual trait for small molecules, and suggests that **1** is able to penetrate the multicellular architecture of osteospheres, and thus could be highly translatable (with respect to in vivo studies). Notably, the potency of **1** towards OSC osteospheres was significantly higher than cisplatin, carboplatin, and salinomycin. Cytotoxicity studies in the presence of various signaling pathway inhibitors suggested that **1** induced OSC death in a COX-2 dependent manner and via necroptosis, but not via apoptosis or random necrosis. Given the potential of **1** to trigger necroptosis, this compound could be further developed to overcome apoptosis resistance in OSCs.

An important distinction between necroptosis and other modes of cell death is that the former leads to the release of signalling molecules called cytokines that can trigger inflammation and serve as ‘find-me’ and ‘destroy-me’ signals for immune cells [37]. Recent studies showed that the induction of necroptosis in cancer models led to the recruitment and activation of cytotoxic T-cells, and thus a robust immune response with complete cancer regression and protection from subsequent tumour re-challenges [38]. Therefore, compounds that can induce necroptosis (as opposed to other non-apoptotic forms of cell death), such as **1**, could be used to initiate an OSC-targeted immune response. Theoretically, **1** could kill OSCs via both cytotoxic (COX-2 related necroptosis) and immunogenic pathways. Overall, this study highlights the therapeutic potential of nickel(II)-polypyridyl compounds containing NSAIDs, particularly as potential drug candidates for osteosarcoma treatments.

## Figures and Tables

**Table 1:** IC_50_ values of **1**, flufenamic acid, cisplatin, carboplatin and salinomycin against U2OS and U2OS-MTX cells and U2OS-MTX osteospheres determined after three or 10 days incubation (mean of two or three independent experiments ± SD).

Compound	U2OS IC_50_ [μM]	U2OS-MTX IC_50_ [μM]	OSC-osteosphere IC_50_ [μM]
**1**	25.16 ± 0.40	26.90 ± 0.71	2.97 ± 0.04
flufenamic acid	>100	>100	13.17 ± 0.24
cisplatin ^1^	16.30 ± 0.50	33.87 ± 3.71	16.49 ± 0.20
carboplatin ^1^	157.50 ± 2.21	114.98 ± 2.31	22.77 ± 0.09
salinomycin ^1^	6.09 ± 1.06	1.49 ± 0.26	4.70 ± 0.08

^1^ Taken from [30].

## Data Availability

Not applicable.

## Supplementary Materials

The following supporting information can be downloaded at: www.mdpi.com/article/10.3390/molecules27103277/s1, Figure S1: ATR-FTIR spectra of the nickel(II)-flufenamic acid complexes **1** (A) and **2** (B); Figure S2: UV-Vis spectra of **1** and **2** (50 μM) in PBS:DMSO (100:1) at room temperature; Figure S3: UV-Vis spectrum of **1** (50 μM) in DMSO over the course of 24 h at 37 °C; Figure S4: UV-Vis spectrum of **2** (50 μM) in DMSO over the course of 24 h at 37 °C; Figure S5: Representative dose-response curves for the treatment of U2OS or U2OS-MTX cells with **1** after 72 h incubation; Figure S6: Representative dose-response curves for the treatment of U2OS or U2OS-MTX cells with flufenamic acid after 72 h incubation; Figure S7: Representative dose-response curves for the treatment of U2OS-MTX osteospheres with **1** or flufenamic acid after 10 days incubation; Figure S8: Representative dose-response curves for the treatment of U2OS-MTX cells with **1** in the absence and presence of z-VAD-FMK (5 µM) or IM-54 (10 µM) after 72 h incubation.

## Sample Availability

Samples of the compounds **1** and **2** are available from the authors.
