# Supplementary material for: An Osteosarcoma Stem Cell Potent Nickel(II)-Polypyridyl Complex Containing Flufenamic Acid
Source: Molecules. 2022 May 19;27(10):3277. doi: 10.3390/molecules27103277 (PMC9143476; doi:10.3390/molecules27103277)
Supplement: Supplementary file 1 [file molecules-27-03277-s001.zip › molecules-1704217-Supplementary.pdf]

Supporting Information for

An Osteosarcoma Stem Cell Potent Nickel(II)-Polypyridyl Complex Containing Flufenamic Acid

Ginevra Passeri [1], Joshua Northcote-Smith [1], Roshane Perera [1], Nikola Gubic [1], and Kogularamanan Suntharalingam [1,*]

[1] School of Chemistry, University of Leicester, Leicester, LE1 7RH, United Kingdom;

* To whom correspondence should be addressed:
Email: k.suntharalingam@leicester.ac.uk

Table of Content

Experimental Details

Figure S1. ATR-FTIR spectra of the nickel(II)-flufenamic acid complexes **1** (A) and **2** (B).

Figure S2. UV-Vis spectra of **1** and **2** (50 μM) in PBS:DMSO (100:1) at room temperature.

Figure S3. UV-Vis spectrum of **1** (50 μM) in DMSO over the course of 24 h at 37 °C.

Figure S4. UV-Vis spectrum of **2** (50 μM) in DMSO over the course of 24 h at 37 °C.

Figure S5. Representative dose-response curves for the treatment of U2OS or U2OS-MTX cells with **1** after 72 h incubation.

Figure S6. Representative dose-response curves for the treatment of U2OS or U2OS-MTX cells with flufenamic acid after 72 h incubation.

Figure S7. Representative dose-response curves for the treatment of U2OS-MTX osteospheres with **1** or flufenamic acid after 10 days incubation.

Figure S8. Representative dose-response curves for the treatment of U2OS-MTX cells with **1** in the absence and presence of z-VAD-FMK (5 μM) or IM-54 (10 μM) after 72 h incubation.

Experimental Details

Materials and Methods. All synthetic procedures were performed under normal atmospheric conditions. Fourier transform infrared (FTIR) spectra were recorded with an IRAffinity-1S Shimadzu spectrophotometer. UV-Vis absorption spectra were recorded on a Cary 3500 UV-Vis spectrophotometer. Elemental analysis of the compounds prepared was performed commercially by the University of Cambridge. $NiCl_2·6H_2O$, 3,4,7,8-tetramethyl-1,10-phenanthroline, 4,7-diphenyl-1,10-phenanthroline, and flufenamic acid were purchased from Sigma Aldrich and used as received.

Synthesis of [Ni(flufenamic acid-*O*)$_2$(3,4,7,8-tetramethyl-1,10-phenanthroline)(H_2O)$_2$] (1): A methanolic solution (15 mL) of flufenamic acid (141 mg, 0.5 mmol) and KOH (28 mg, 0.5 mmol) was stirred for 1 h. To the resulting solution, a methanolic solution (5 mL) of $NiCl_2·6H_2O$ (59 mg, 0.25 mmol) was added simultaneously with a methanolic solution (15 mL) of 3,4,7,8-tetramethyl-1,10-phenanthroline (59 mg, 0.25 mmol). The resulting solution was stirred for 16 h before being fully dried. The resulting solid was washed with water (10 mL) and diethyl ether (1 mL) to yield **1** as a pale green solid (43 mg, 19%). UV (PBS:DMSO (100:1), nm): 277, 300, 340; ATR-FTIR (solid, cm^{-1}): 3253, 3072, 2976, 1583, 1506, 1456, 1378, 1329, 1291, 1247, 1158, 1120, 1070, 999, 932, 888, 861, 811, 795, 750, 723, 695, 662, 624, 574, 524, 458, 414; Anal. Calcd. For **1**, $C_{44}H_{38}F_6N_4NiO_6$ (%): C, 59.28; H, 4.30; N, 6.28. Found: C, 59.26; H, 3.96; N, 6.40.

Synthesis of [Ni(flufenamic acid-*O*)(flufenamic acid-*O,O'*)(4,7-diphenyl-1,10-phenanthroline)(H_2O)] (2): A methanolic solution (15 mL) of flufenamic acid (141 mg, 0.5 mmol) and KOH (28 mg, 0.5 mmol) was stirred for 1 h. To the resulting solution, a methanolic solution (5 mL) of $NiCl_2·6H_2O$ (59 mg, 0.25 mmol) was added simultaneously with a methanolic solution (15 mL) of 4,7-diphenyl-1,10-phenanthroline (83 mg, 0.25 mmol). The resulting solution was stirred for 16 h before being fully dried. The resulting solid was washed with water (10 mL) and diethyl ether (1 mL) to yield **2** as a pale green solid (120 mg, 50%). UV (PBS:DMSO (100:1), nm): 288, 337; ATR-FTIR (solid, cm^{-1}): 3251, 3070, 2985, 1581, 1509, 1459, 1390, 1329, 1287, 1233, 1158, 1120, 1070, 996, 929, 858, 845, 812, 791, 753, 699, 665, 632, 578, 548, 523, 452, 414; Anal. Calcd. For **2**, $C_{52}H_{36}F_6N_4NiO_5$ (%): C, 64.42; H, 3.74; N, 5.78. Found: C, 64.55; H, 3.56; N, 5.81.

Measurement of water-octanol partition coefficient (LogP). The LogP value for **1** and **2** was determined using the shake-flask method and UV-Vis spectroscopy. The octanol used in this experiment was pre-saturated with water. An aqueous solution of **1** and **2** (500 µL, 100 µM) was incubated with octanol (500 µL) in a 1.5 mL tube. The tube was shaken at room temperature for 24 h. The two phases were separated by centrifugation and the **1** and **2** content in each phase was determined by UV-Vis spectroscopy.

Cell Lines and Cell Culture Conditions. The U2OS bone osteosarcoma cell line was acquired from American Type Culture Collection (ATCC, Manassas, VA, USA) and cultured in Dulbecco's Modified Eagle's Medium (DMEM) supplemented with 10% fetal bovine serum and 1% penicillin. The cells were grown at 310 K in a humidified atmosphere containing 5% CO_2. To gain access to osteosarcoma stem cell (OSC)-enriched cells, a full T75 flask of U2OS cells was treated with methotrexate (300 nM) for 4 days. The cells (labelled U2OS-MTX cells) were used immediately. U2OS-MTX cells were characterised according to CD117 expression using flow cytometry as previously reported [Robin, P.; Singh, K.; Suntharalingam, K. *Chem. Commun.* **2020**, *56*, 1509-1512].

Cytotoxicity MTT assay. The colourimetric MTT assay was used to determine the toxicity of **1** and flufenamic acid. U2OS and U2OS-MTX cells (5×10^3) were seeded in each well of a 96-well plate. After incubating the cells overnight, various concentrations of the compounds (0.2-100 μM), were added and incubated for 72 h (total volume 200 μL). Stock solutions of the compounds were prepared as 10 mM solutions in DMSO and diluted using media. The final concentration of DMSO in each well was 0.5% and this amount was present in the untreated control as well. After 72 h, 20 μL of a 4 mg/mL solution of MTT in PBS was added to each well, and the plate was incubated for an additional 4 h. The DMEM/MTT mixture was aspirated and 100 μL of DMSO was added to dissolve the resulting purple formazan crystals. The absorbance of the solutions in each well was read at 550 nm. Absorbance values were normalized to (DMSO-containing) control wells and plotted as concentration of test compound versus % cell viability. IC_{50} values were interpolated from the resulting dose dependent curves. The reported IC_{50} values are the average of three independent experiments, each consisting of six replicates per concentration level (overall n = 18).

Osteosphere Formation and Viability Assay. U2OS-MTX cells (5×10^3) were plated in ultralow-attachment 96-well plates (Corning) and incubated in DMEM/F12 medium supplemented with N2 (Invitrogen), human EGF (10 ng/mL), and human bFGF (10 ng/mL) for 10 days. Studies were also conducted in the presence of **1** and flufenamic acid (0-133 μM). Osteospheres treated with **1** and flufenamic acid (at their respective IC_{20} values, 10 days) were imaged using an inverted microscope. The viability of the osteospheres was determined by addition of a resazurin-based reagent, TOX8 (Sigma). After incubation for 16 h, the fluorescence of the solutions was read at 590 nm (λ_{ex} = 560 nm). Viable osteospheres reduce the amount of the oxidized TOX8 form (blue) and concurrently increase the amount of the fluorescent TOX8 intermediate (red), indicating the degree of osteosphere cytotoxicity caused by the test compound. Fluorescence values were normalized to DMSO-containing controls and plotted as concentration of test compound versus % osteosphere viability. IC_{50} values were interpolated from the resulting dose dependent curves. The reported IC_{50} values are the average of two independent experiments, each consisting of two replicates per concentration level (overall n = 4).

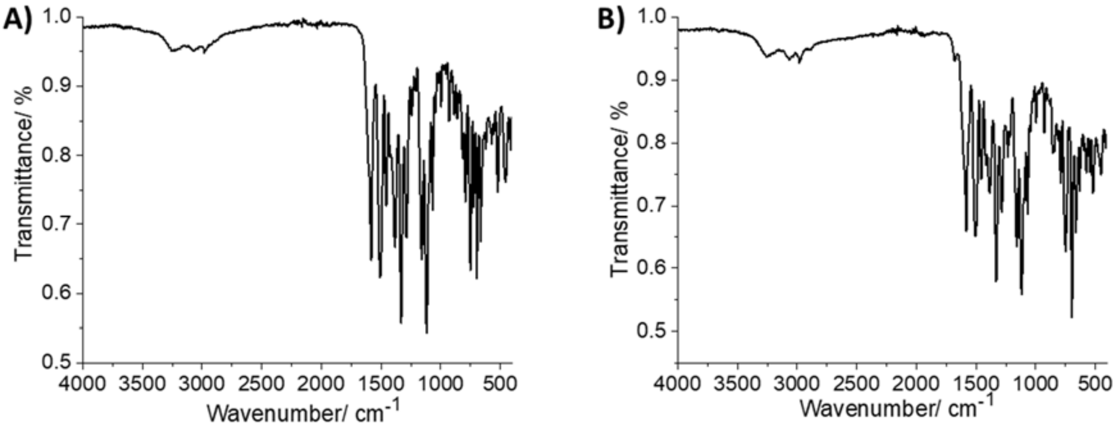

Figure S1. ATR-FTIR spectra of the nickel(II)-flufenamic acid complexes **1** (A) and **2** (B).

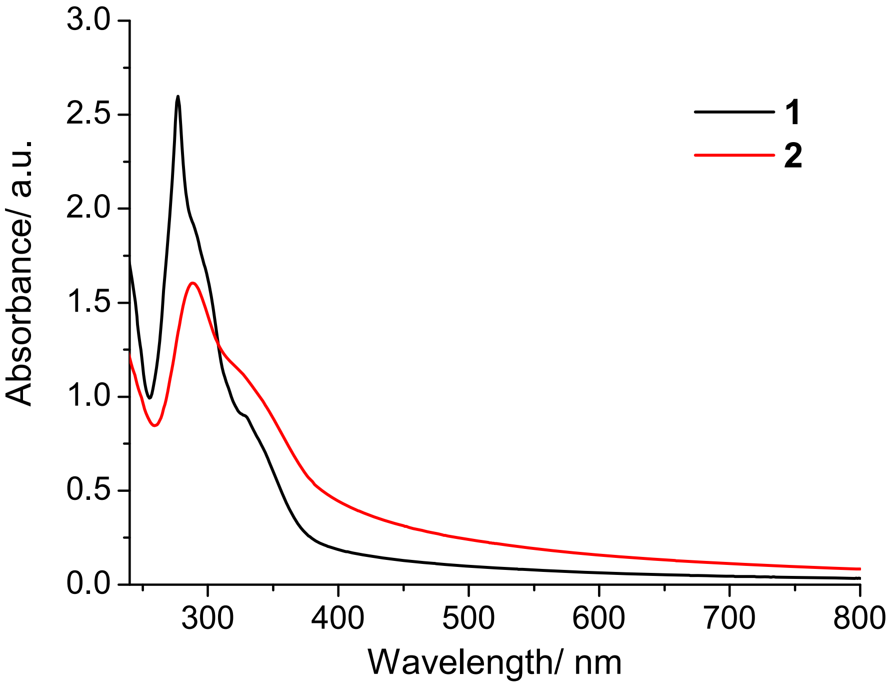

Figure S2. UV-Vis spectra of **1** and **2** (50 μM) in PBS:DMSO (100:1) at room temperature.

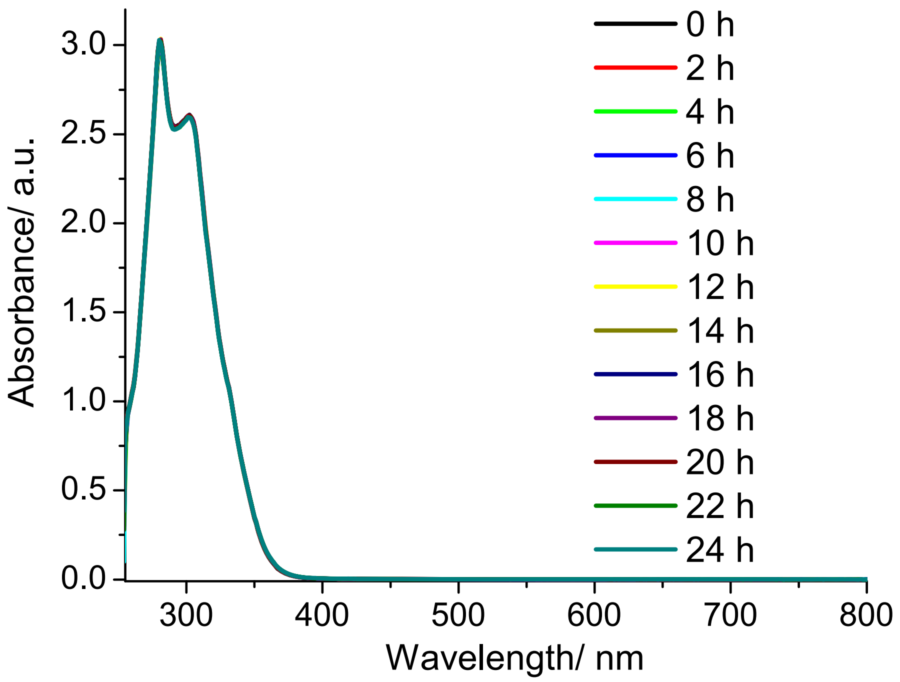

Figure S3. UV-Vis spectrum of **1** (50 μM) in DMSO over the course of 24 h at 37 °C.

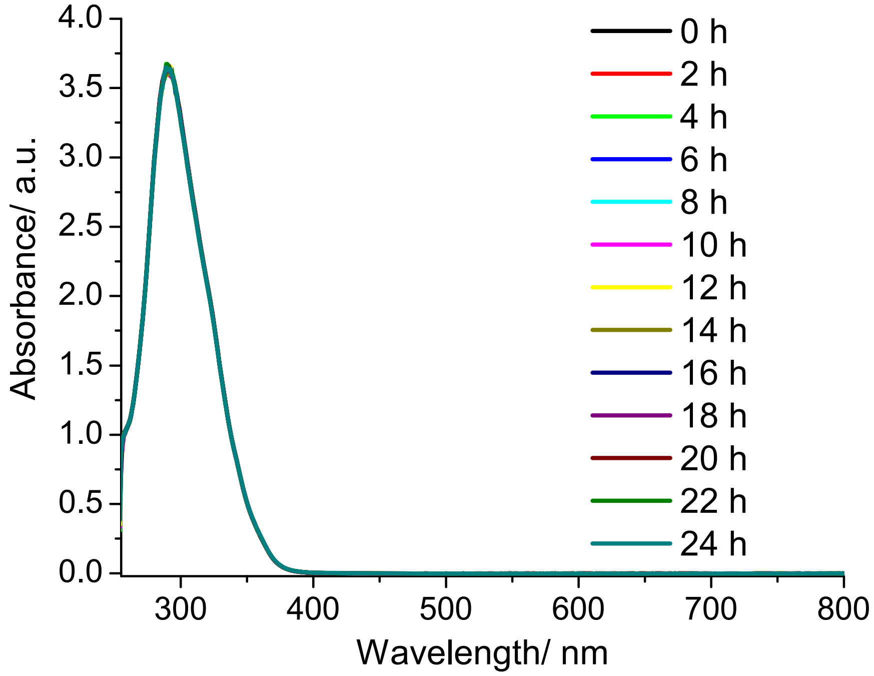

Figure S4. UV-Vis spectrum of **2** (50 μM) in DMSO over the course of 24 h at 37 °C.

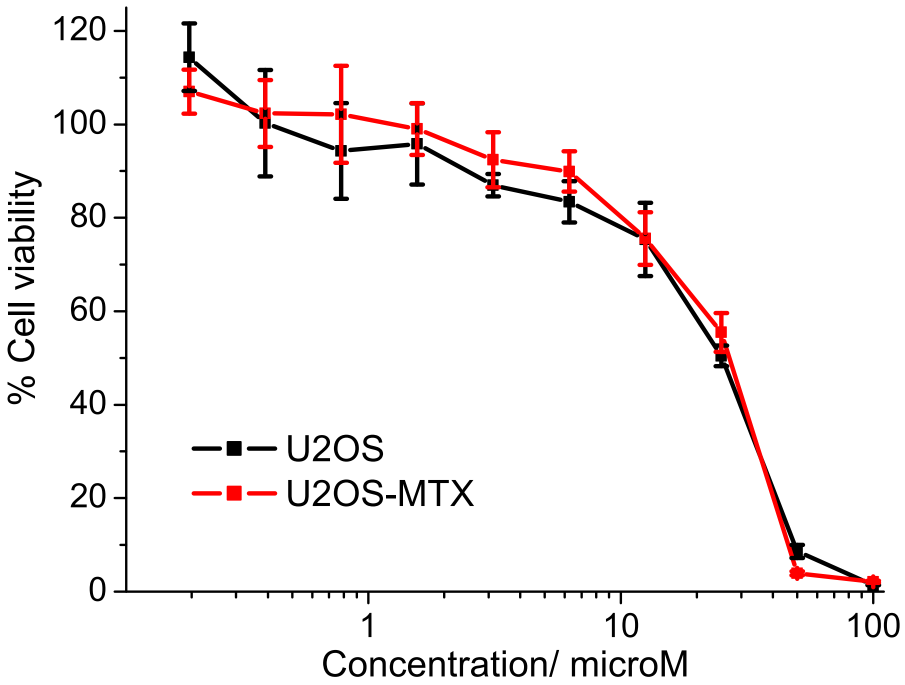

Figure S5. Representative dose-response curves for the treatment of U2OS or U2OS-MTX cells with **1** after 72 h incubation.

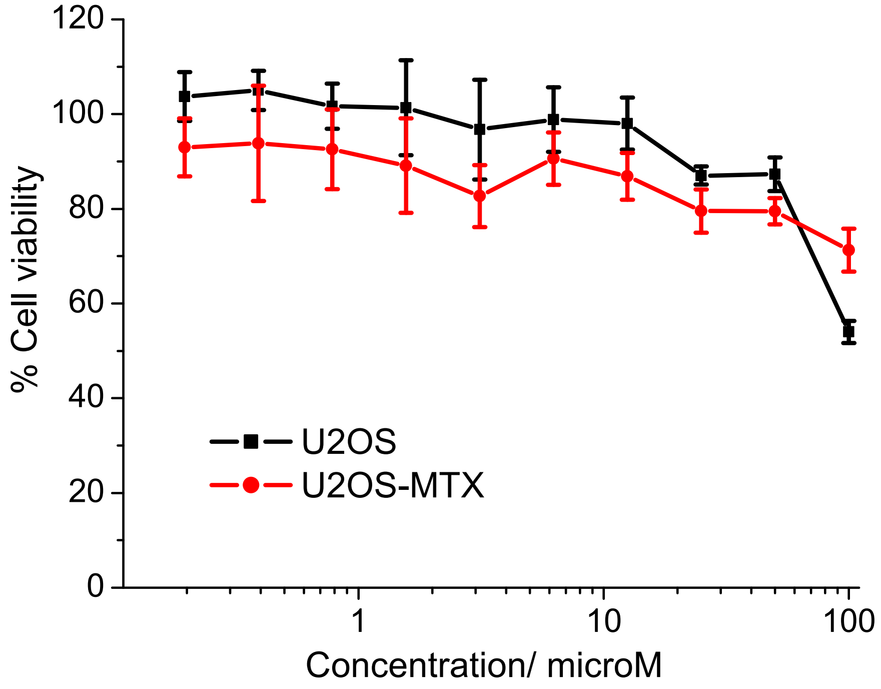

Figure S6. Representative dose-response curves for the treatment of U2OS or U2OS-MTX cells with flufenamic acid after 72 h incubation.

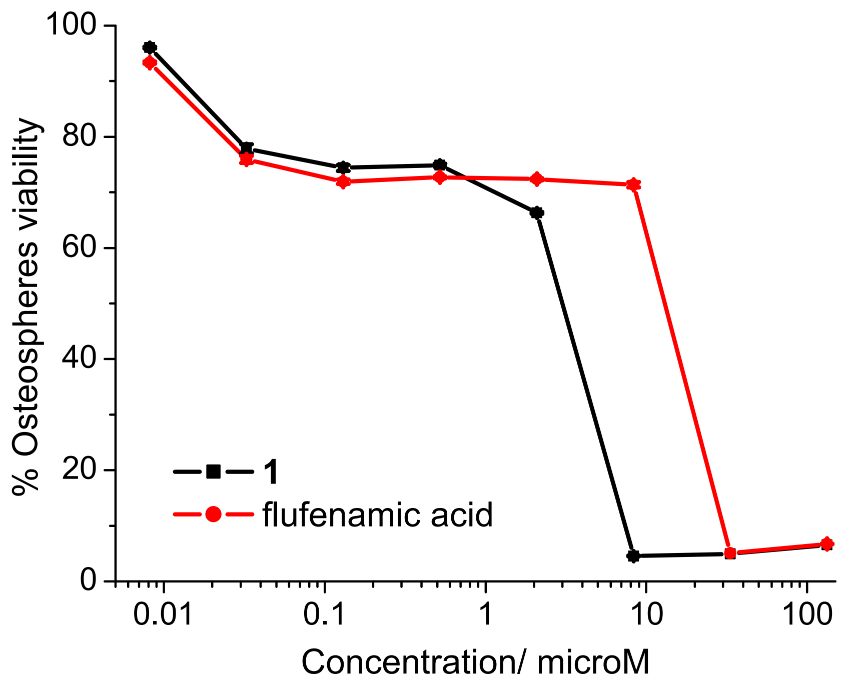

Figure S7. Representative dose-response curves for the treatment of U2OS-MTX osteospheres with **1** or flufenamic acid after 10 days incubation.

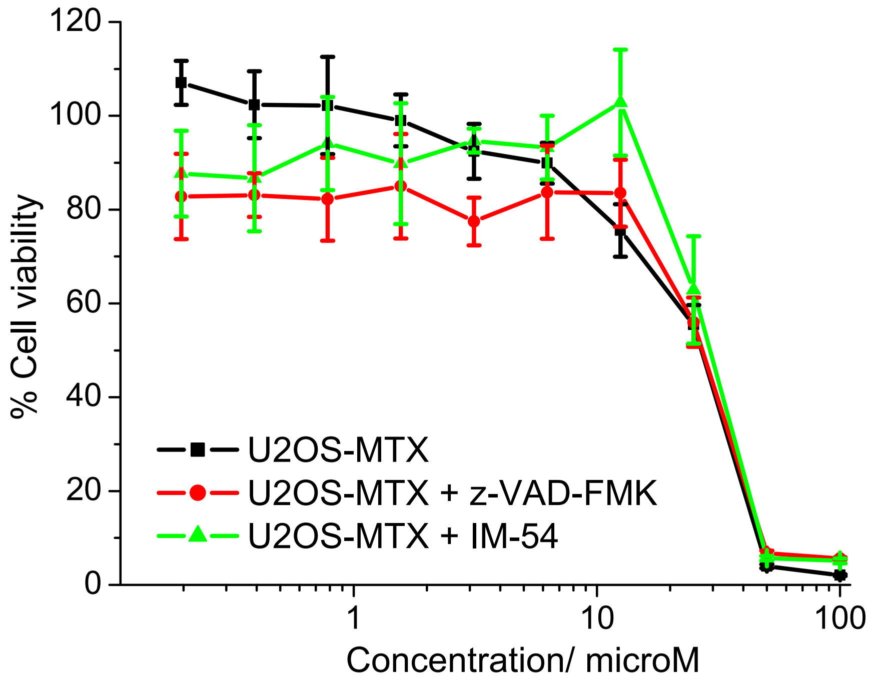

Figure S8. Representative dose-response curves for the treatment of U2OS-MTX cells with **1** in the absence and presence of z-VAD-FMK (5 μM) or IM-54 (10 μM) after 72 h incubation.
